# Globular C1q Receptor (gC1qR/p32/HABP1) Is Overexpressed in Malignant Pleural Mesothelioma and Is Associated With Increased Survival in Surgical Patients Treated With Chemotherapy

**DOI:** 10.3389/fonc.2019.01042

**Published:** 2019-10-11

**Authors:** Xiaoyu Li, Takashi Eguchi, Rania G. Aly, Navin K. Chintala, Kay See Tan, Marjorie G. Zauderer, Francine R. Dembitzer, Mary Beth Beasley, Berhane Ghebrehiwet, Prasad S. Adusumilli, Ellinor I. B. Peerschke

**Affiliations:** ^1^Thoracic Service, Department of Surgery, Memorial Sloan Kettering Cancer Center, New York, NY, United States; ^2^Department of Thoracic Oncology, West China Hospital, Sichuan University, Chengdu, China; ^3^Division of Thoracic Surgery, Department of Surgery, Shinshu University, Matsumoto, Japan; ^4^Department of Pathology, Memorial Sloan Kettering Cancer Center, New York, NY, United States; ^5^Department of Pathology, Alexandria University, Alexandria, Egypt; ^6^Department of Epidemiology and Biostatistics, Memorial Sloan Kettering Cancer Center, New York, NY, United States; ^7^Thoracic Oncology Service, Division of Solid Tumor Oncology, Department of Medicine, Memorial Sloan Kettering Cancer Center, New York, NY, United States; ^8^Department of Pathology, Mount Sinai School of Medicine, New York, NY, United States; ^9^Department of Medicine, Stony Brook University School of Medicine, Stony Brook, NY, United States; ^10^Center for Cell Engineering, Memorial Sloan Kettering Cancer Center, New York, NY, United States; ^11^Department of Laboratory Medicine, Memorial Sloan Kettering Cancer Center, New York, NY, United States

**Keywords:** gC1qR/p32/HABP1 (gC1qR), malignant pleural mesothelioma, chemotherapy, CD4 T cell, complement system

## Abstract

**Introduction:** Globular C1q receptor (gC1qR/p32/HABP1) is overexpressed in a variety of cancers, particularly adenocarcinomas. This study investigated gC1qR expression in malignant pleural mesothelioma (MPM) and its pathophysiologic correlates in a surgical patient cohort.

**Methods:** Tissue microarrays comprising 6 tumoral and 3 stromal cores from 265 patients with MPM (216 epithelioid, 26 biphasic, and 23 sarcomatoid; 1989–2010) were investigated by immunohistochemistry for gC1qR expression (intensity and distribution by H-score, range 0–300), and immune cell infiltration. Overall survival (OS) was analyzed by the Kaplan-Meier method (high vs. low gC1qR expression delineated by median score) in the whole cohort and by neoadjuvant chemotherapy (NAC) status. Multivariable Cox analysis included stage, chemotherapy, and immune cell infiltration.

**Results:** gC1qR was overexpressed in all histological types of MPMs (263/265, 99.2%) compared to normal pleura. In epithelioid MPM, high gC1qR expression was associated with better OS (median 25 vs. 11 months; *p* = 0.020) among NAC patients, and among patients without NAC (No-NAC) but who received post-operative chemotherapy (median OS 38 vs. 19 months; *p* = 0.0007). In multivariable analysis, high gC1qR expression was an independent factor for improved OS in patients treated with NAC. In the No-NAC cohort, high gC1qR expression correlated with lower tumor stage. Moreover, the influence of Ki67 and CD4 T-cell infiltration on OS were more pronounced among patients with high gC1qR expression.

**Conclusion:** This is the first description of gC1qR expression in MPM. The data identify gC1qR as a potential new prognostic factor in patients treated with surgery and chemotherapy.

## Introduction

The complement system, particularly subcomponent C1q, plays major role in innate and adaptive immunity (1). C1q interacts with immune complexes to activate complement and generate inflammatory mediators. It is also involved in the clearance of apoptotic cell debris and B cell tolerance (2), regulation of T cell proliferation and cytokine expression (1, 3), as well as regulation of monocyte-derived dendritic cell differentiation (4).

gC1qR/p32/HABP1 (gC1qR) binds the globular domain of C1q and is a multicompartmental and multiligand binding cellular protein (4–7). It is expressed on the cell surface, mitochondria, cytosol, and the extracellular microenvironment (8–11). Expanding non-immune functions of gC1R have been identified in recent years, including its participation in cancer. In adenocarcinoma, gC1qR has been shown to play a role in tumor cell proliferation, migration, and immune modulation (12). gC1qR is highly expressed by proliferating cells, and is upregulated in carcinomas (13, 14). In a series of small studies, overexpression of gC1qR has been associated with poor prognosis in patients with prostate, breast, serous ovarian, and endometrial cell cancers (15–18).

Intracellular gC1qR has been found to be associated with chemotherapy induced apoptosis. In cervical cancer, gC1qR transcription was upregulated *in vitro* following cisplatin treatment of tumor cells and was associated with cisplatin-induced apoptosis (19). Similarly, paclitaxel treated ovarian cancer cells showed increased gC1qR expression associated with cell apoptosis and mitochondrial dysfunction (20).

On the cell surface, gC1qR binds to variety of ligands linked to immune modulation and inflammation (21, 22). For example, gC1qR plays a pivotal role in the regulation of antiviral T cell responses and in compromising CD4 T cell function (23). In addition, gC1qR has been linked to immune evasion (5) and cell proliferation in adenocarcinoma of the breast (24, 25). gC1qR expression in mesothelioma has not been studied.

Malignant pleural mesothelioma (MPM) is a rare and aggressive cancer, typically associated with asbestos exposure (26, 27) Treatment outcomes continue to be poor with a median survival, of ~12 months (28). For patients with the epithelioid subtype who underwent trimodality therapy, which includes surgery, chemotherapy, and radiation, median survival is extended to 23.4 months (29). The application of pemetrexed/cisplatin in MPM provides a response rate of about 40% (30), but there is no marker available to stratify patients to chemotherapy in MPM.

This study examined the expression of gC1qR in 265 cases of MPM, including epithelioid (*n* = 216), sarcomatoid (*n* = 23), and biphasic (*n* = 26) histiologic subtypes. Since immunologic markers are increasingly recognized as important prognostic indicators in cancer and may predict treatment efficacies, significant correlations between gC1qR expression and patient clinicopathologic characteristics were investigated.

## Materials and Methods

### Patients

This retrospective study was approved by the Institutional Review Board (WA-0436-10) of Memorial Sloan Kettering Cancer Center (MSK). A total of 620 cases of MPM diagnosed at MSK between 1989 and 2010 were reviewed. From this cohort, 395 MPM cases had available hematoxylin and eosin (H&E)-stained slides. All slides were re-evaluated by two pathologists (31) yielding 301 epithelioid, 59 biphasic, and 35 sarcomatoid MPMs. Of these, 283 patients had tumor blocks available for the construction of tissue microarrays (TMAs). Median follow-up was 16 months (range 0–187 months). Clinical data were collected from the prospectively maintained MPM database. Patients with mesotheliomas either underwent surgical resection without neoadjuvant chemotherapy (No-NAC cohort) or received NAC (NAC cohort) prior to resection. Most patients underwent extrapleural pneumonectomy (EPP) or pleurectomy with decortication (PD), as shown in Table 1. There was no statistical difference between type of surgical tumor resection, comparing No-NAC and NAC groups (Table 2). Patients were not stratified further according to surgical procedure, given equivalent outcomes between EPP and PD surgeries (32).

**Table 1:** Demographics and clinicopathologic characteristics of patients with epithelioid and non-epithelioid MPM.

		**Epithelioid**	**Non-epithelioid**
***N* = 265**		***N* = 216 (%)**	***N* = 49 (%)**
Age		63 (54–69)	66 (62–73)
Sex	Female	63 (29)	6 (12)
	Male	153 (71)	43 (88)
Smoking status (*n* = 203)	(–)	44 (26)	8 (22)
	(+)	123 (74)	28 (78)
Asbestos (*n* = 187)	(–)	65 (42)	7 (21)
	(+)	89 (58)	26 (79)
Procedure	EPP	123 (57)	19 (39)
	PD	81 (38)	23 (47)
	Other	12 (6)	7 (14)
R status (*n* = 254)	R1	174 (81)	29 (59)
	R2	31 (14)	20 (41)
Chemotherapy status			
Neoadjuvant chemotherapy followed by surgery		59 (27)	8 (16)
No neoadjuvant chemotherapy		157 (73)	41 (84)
Any chemotherapy after surgery		38 (18)	2 (4)
No chemotherapy after surgery		88 (41)	37 (76)
Unknown chemotherapy status after surgery		31 (14)	2 (4)
p-Stage (*n* = 264)	I	10 (5)	0 (0)
	II	59 (27)	8 (17)
	III	124 (57)	28 (58)
	IV	23 (11)	12 (25)
T category (*n* = 263)	T1	14 (7)	0 (0)
	T2	95 (44)	12 (25)
	T3	89 (41)	25 (52)
	T4	17 (8)	11 (23)
N category (*n* = 257)	N0	150 (71)	38 (93)
	N1	15 (7)	1 (2)
	N2	47 (22)	5 (12)
	N3	0 (0)	1 (2)
gC1qR expression	(–)	2 (1)	0 (0)
	(+)	214 (99)	49 (100)
gC1qR H-score		156 (85–206)	150 (111–188)

*Data are number (%) or median (25 and 75 percentiles). gC1qR, globular heads of the C1q receptor*.

**Table 2:** Demographics and clinicopathologic demographics of patients with epithelioid MPM: comparison between patients treated with or without neoadjuvant chemotherapy (NAC).

		**No NAC**	**NAC**	
***N* = 216**		***N* = 157**	***N* = 59**	***P***
Age		63 (56–70)	58 (50–67)	**0.004**
Sex	Female	45 (29)	18 (31)	0.9
	Male	112 (71)	41 (69)	
Smoking status (*n* = 167)	(–)	28 (25)	16 (28)	0.7
	(+)	82 (75)	41 (72)	
Asbestos (*n* = 154)	(–)	37 (37)	28 (53)	0.060
	(+)	64 (63)	25 (47)	
Procedure	EPP	85 (54)	38 (64)	0.4
	PD	63 (40)	18 (31)	
	Other	9 (6)	3 (5)	
R status (*n* = 205)	R1	126 (84)	48 (87)	0.7
	R2	24 (16)	7 (13)	
p-Stage	I	6 (4)	4 (7)	0.5
	II	40 (25)	19 (32)	
	III	93 (59)	31 (53)	
	IV	18 (11)	5 (8)	
T category (*n* = 215)	T1	10 (6)	4 (7)	1.0
	T2	69 (44)	26 (44)	
	T3	65 (42)	24 (41)	
	T4	12 (8)	5 (8)	
N category (*n* = 212)	N0	104 (67)	46 (81)	0.089
	N1	14 (9)	1 (2)	
	N2	37 (24)	10 (18)	
Pleomorphic morphology	(–)	133 (85)	48 (81)	0.5
	(+)	24 (15)	11 (19)	
Lymphatic invasion (*n* = 215)	(–)	78 (50)	29 (49)	1.0
	(+)	78 (50)	30 (51)	
Vascular invasion (*n* = 215)	(–)	118 (76)	44 (75)	0.9
	(+)	38 (24)	15 (25)	
Ki-67 index (%) (*n* = 212)		9.2 (3.8, 17.5)	8.1 (3.4, 12.9)	0.4
gC1qR expression	(–)	2 (1)	0 (0)	
	(+)	155 (99)	59 (100)	
gC1qR H-score (all)		151 (80, 200)	166 (99, 229)	0.2
gC1qR H-score (no post-op chemo) (*n* = 88)		149 (86, 202)		
gC1qR H-score (post-op chemo) (*n* = 38)		163 (80, 205)		

*Data are number (%) or median (25 and 75 percentiles). MPM, malignant pleural mesothelioma; NAC, neoadjuvant chemotherapy; EPP, extrapleural pneumonectomy; PD, pleurectomy/decortication; gC1qR, globular heads of the C1q receptor. Bold value indicates significant p-value*.

### Tissue Microarrays

Formalin-fixed, paraffin-embedded tumor blocks were used for the construction of TMAs. Six to nine representative tumor areas with the most abundant inflammatory reaction were marked on H&E slides (31, 33). For biphasic tumors, tumor areas were selected from a predominantly sarcomatoid area. Cylindrical 0.6 mm tissue cores were arrayed from the marked areas of corresponding paraffin blocks onto a recipient block using an automated tissue arrayer (ATA-27; Beecher Instruments, Sun Prairie, WI).

### Histologic Evaluation

Histologic evaluation was performed by pathologists using an Olympus BX51 microscope (Olympus Optical Co. Ltd., Tokyo, Japan) with a standard 22-mm diameter eyepiece (31). Tumors were classified as either epithelioid, sarcomatoid, or biphasic according to the 2015 World Health Organization classification (34). Epithelioid MPMs were further classified as pleomorphic subtype when cytologic pleomorphisms accounted for ≥10% of the tumor.

The distribution of tumor area and tumor-associated stroma was determined in each core. To evaluate tumor infiltrating immune cells, cores with ≥ 50% of tumor-associated stroma were excluded from the analysis to decrease the confounding bias of tumor infiltrating immune cells from the stroma (35, 36).

### Immunohistochemical Staining

Paraffin 4 μm-thick sections were cut from the TMA blocks and deparaffinized. Sections stained with the gC1qR primary antibody (clone 60.11, 1 μg/ml) were incubated for 2 h at room temperature followed by incubation with the biotinylated secondary for 10 min (14, 37). DAB was used for visualization and hematoxylin for nuclear counter stain. Standard avidin-biotin-peroxidase complex technique was used for immunohistochemical staining for CD3, CD4, CD8, CD20, CD68, CD163, Foxp3, and Ki-67, as previously described (31, 38).

Normal pleura served as controls (*n* = 6). Specimens were obtained from surgical samples of patients undergoing thoracic surgery during which pleural samples were taken. Final pathology confirmed normal pleura and no malignancy.

### Scoring of Immunohistochemical Staining

Expression of gC1qR was mainly observed in the cytoplasm of tumor cells. We evaluated the overexpression of gC1qR by H score, which included the intensity and percentage of positive tumor cells (31, 38). The intensity of gC1qR expression was determined by pathologists as follows: 0 for no expression, 1 for weak, 2 for moderate, and 3 for strong. The distribution of tumor cells with each intensity among all tumor cells was also recorded. H score was assigned to each core based on the intensity and the distribution of tumor cells with each intensity (H score = 1 × [% of tumor cells with weak intensity] + 2 × [% of tumor cells with moderate intensity] + 3 × [% of tumor cells with strong intensity]; range 0–300). Individual H scores in multiple cores from same tumor were averaged to obtain a single H score for each patient. Ki-67 proliferation index was recorded as the percentage of tumor cells with nuclear positive immunostaining in each tissue microarray core (38). Tumor infiltrating immune cells were counted in each core and scored as previously described (31). Immunohistochemical evaluation was performed by two independent pathologists. In 95% of cases, H scores were the same between pathologists. In the remaining cases, the two scores were averaged.

### Chemotherapy

In 216 epithelioid MPMs, 59 patients underwent chemotherapy prior to surgical resection (neoadjuvant chemotherapy [NAC] cohort, *n* = 59). In this group, in addition to NAC, 3 patients received adjuvant chemotherapy, 16 received chemotherapy following recurrence, 43 received radiation therapy. In patients who did not receive chemotherapy prior to surgical resection (no-NAC cohort, *n* = 157), 17 received adjuvant chemotherapy, 21 received chemotherapy following recurrence, and 77 patients received radiation therapy. Because the treatment regimens were variable, the study cohort was broadly divided into 2 groups, NAC and no-NAC. Due to the focus on clinical relevance, the impact of gC1qR on epithelioid MPM was investigated separately in NAC and no-NAC group as an a-priori decision.

### Statistical Analysis

The gC1qR expression, IHC score of the tumor infiltrating immune cells, and Ki-67 index were dichotomized into high and low, using median values identified in each cohort (epithelioid NAC, epithelioid no NAC). The association between clinicopathologic factors and gC1qR expression was analyzed by Fisher's exact test for categorical variables and Wilcoxon rank sum test for continuous variables. The primary endpoint was overall survival (OS), defined from the time of surgery to the time of death from any cause, and otherwise censored at the date of last follow up. In this surgical cohort, overall 30 and 90 day mortality of patients with epitheloid mesothelioma was 4% (8/216) and 9% (20/216), respectively. All patients were included in the survival analysis, and patients who died in these time frames were considered as death events in the overall survival analysis. OS was estimated by the Kaplan-Meier method and compared between groups using the log-rank test, stratified by stage. Association between factors and death were quantified by Cox proportional hazards regression models. Variables with significant interaction were evaluated in combination with gC1qR for prognostic analysis. Multivariable modeling was conducted using a backward selection process, starting with factors with *p* < 0.1 in univariable analyses. The proportional hazards assumption was assessed through Schoenfeld residuals; there was no evidence of violation of the proportionality assumption. Combination variables included gC1qR expression combined with CD4 T cell infiltration (low gC1qR with low CD4; low-high; high-low and high levels of both), and gC1qR expression combined with Ki-67 expression (low gC1qR with low Ki67; low-high; high-low and high levels of both). All analyses were conducted using Stata 13.1 (Stata Corp, College Station, TX) and R 3.5.3 (R Core Team, Vienna, Austria). Analyses were two-sided and *p*-values < 0.05 were considered significant.

## Results

### gC1qR Expression in Epithelioid and Non-epithelioid MPM: gC1qR Is Expressed in Epitheloid and Non-epitheliod MPM Subtypes

**Figure 1:**
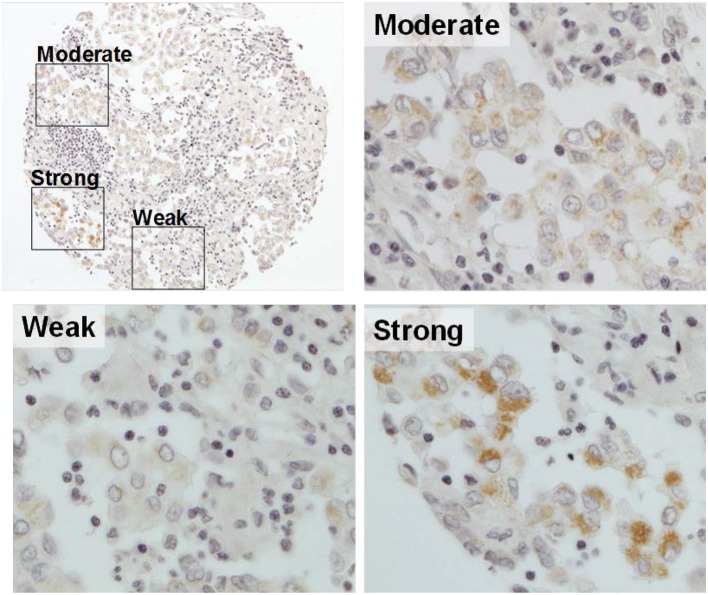
Representative image of gC1qR expression in a tumor core. A single MPM tumor core with immunohistochemical staining of gC1qR demonstrates heterogeneous expression—weak staining (10% of the tumor core); moderate staining (80%); strong staining (10%). H score of gC1qR for this core is 200 (10 × 1+80 × 2+10 × 3) (original magnification upper left X10, others X20).

IHC analysis of gC1qR expression was performed using TMA from 265 patient tumors (range of cores per patient: 1–9, median number of cores: 6; <5% (13/265) of tumors had a single core). Patient demographics and clinicopathologic information are presented in Table 1. Most patients (*n* = 216, 82%) were diagnosed with epithelioid MPM. The remaining 49 patients were diagnosed with non-epithelioid: 26 biphasic and 23 sarcomatoid subtypes. Expression of gC1qR was noted in both epithelioid and non-epithelioid MPM subtypes (positive in 99% of all cases). Expression of gC1qR was heterogeneous within an individual tumor and across tumors from different patients. A representative image demonstrating heterogeneous cytoplasmic staining for gC1qR is shown in Figure 1. The median H score for gC1qR staining was 156 (25th, 75th percentiles: 85, 206) for epithelioid MPMs and 150 (111, 188) for non-epithelioid MPMs. There was no statistical difference in gC1qR H scores between epithelioid and non-epithelioid cases (*p* = 0.9). Minimal gC1qR expression was observed in normal pleura (median H score: 30). Due to the small number of non-epithelioid cases in the study set, the remaining analyses focused only on epithelioid cases.

### gC1qR Expression by Chemotherapy Status in Epithelioid MPM: gC1qR Expression Is Similar in NAC and No-NAC Cohorts and Is Not Associated With Tumoral Immune Cell Infiltration

The expression of gC1qR in epithelioid MPM (*n* = 216) was further evaluated by comparing H scores for tumors resected from patients who received neoadjuvant chemotherapy (NAC, *n* = 59) and those who did not (no-NAC, *n* = 157) (Table 2). These patient cohorts did not differ significantly by patient age, sex, smoking status, asbestos exposure, surgical procedure, R status, p-stage, T-category, N-category, pleomorphic tumor morphology, lymphatic invasion, vascular invasion, or Ki-67 index. here was no significant difference in gC1qR expression between NAC and no-NAC groups (median H score in NAC vs. no-NAC; 166 vs. 151, *p* = 0.2). In addition, gC1qR H scores were also not significantly different among patients who did (*n* = 38) or did not (*n* = 88) receive post-operative chemotherapy in the no-NAC cohort (median H score 163 vs. 149; *p* = 1.0).

Table 3 reports patient demographics and clinicopathological characteristics including tumor infiltrating immune cell scores for epithelioid MPMs expressing high and low levels of gC1qR in no-NAC and NAC groups. Higher gC1qR expression was associated with lower stage in the no-NAC group (p-Stage, *p* = 0.040; T category, *p* = 0.005). A similar trend was observed in the NAC cohort, but this did not reach statistical significance (p-Stage, *p* = 0.078; T category, *p* = 0.075). Expression of gC1qR was not associated with other clinicopathologic characteristics such as pleomorphic tumor morphology, lymphatic invasion, vascular invasion, Ki-67 index, or tumor infiltrating immune cells in either no-NAC or NAC groups.

**Table 3:** Comparison of demographics and clinicopathologic characteristics of patients with epitheloid MPM and low or high gC1qR expression in no neoadjuvant chemotherapy (no-NAC) and neoadjuvant chemotherapy (NAC) cohorts.

	**Cohort**	**No-NAC**			**NAC**		
	**gC1qR**	**Low (<151)**	**High (≥151)**		**Low (<166)**	**High (≥166)**	
		***N* = 78**	***N* = 79**	***P***	***N* = 29**	***N* = 30**	***P***
Age		62 (55–68)	65 (56–73)	0.084	58 (52–67)	59 (47–67)	0.7
Gender	Female	26 (33)	19 (24)	0.2	6 (21)	12 (40)	0.2
	Male	52 (67)	60 (76)		23 (79)	18 (60)	
Smoking status (*n* = 167)	(–)	12 (26)	16 (25)	1.0	8 (29)	8 (28)	1.0
	(+)	35 (74)	47 (75)		20 (71)	21 (72)	
Asbestos (*n* = 154)	(–)	18 (43)	19 (32)	0.3	15 (56)	13 (50)	0.8
	(+)	24 (57)	40 (68)		12 (44)	13 (50)	
Procedure	EPP	38 (49)	47 (59)	0.3	17 (59)	21 (70)	0.5
	PD	34 (44)	29 (37)		11 (38)	7 (23)	
	Other	6 (8)	3 (4)		1 (3)	2 (7)	
R status (*n* = 205)	R1	58 (78)	68 (89)	0.077	24 (86)	24 (89)	1.0
	R2	16 (22)	8 (11)		4 (14)	3 (11)	
p-Stage	I	1 (1)	5 (6)	**0.040**	4 (14)	0 (0)	0.078
	II	14 (18)	26 (33)		8 (28)	11 (37)	
	III	53 (68)	40 (51)		13 (45)	18 (60)	
	IV	10 (13)	8 (10)		4 (14)	1 (3)	
T category (*n* = 215)	T1	2 (3)	8 (10)	**0.005**	4 (14)	0 (0)	0.075
	T2	27 (35)	42 (54)		11 (38)	15 (50)	
	T3	42 (54)	23 (29)		10 (34)	14 (47)	
	T4	7 (9)	5 (6)		4 (14)	1 (3)	
N category (*n* = 212)	N0	49 (64)	55 (71)	0.5	24 (83)	22 (79)	0.9
	N1	9 (12)	5 (6)		0	1 (4)	
	N2	19 (25)	18 (23)		5 (17)	5 (18)	
Pleomorphic morphology	(–)	65 (83)	68 (86)	0.7	23 (79)	25 (83)	0.7
	(+)	13 (17)	11 (14)		6 (21)	5 (17)	
Lymphatic invasion (*n* = 215)	(–)	41 (53)	37 (47)	0.6	16 (55)	13 (43)	0.4
	(+)	37 (47)	41 (53)		13 (45)	17 (57)	
Vascular invasion (*n* = 215)	(–)	58 (74)	60 (77)	0.9	25 (86)	19 (63)	0.072
	(+)	20 (26)	18 (23)		4 (14)	11 (37)	
CD3+ T cells (*n* = 212)	Low	30 (39)	37 (47)	0.3	16 (55)	13 (45)	0.6
	High	46 (61)	41 (53)		13 (45)	16 (55)	
CD4+ T cells (*n* = 213)	Low	33 (43)	38 (49)	0.5	14 (48)	15 (52)	1.0
	High	44 (57)	40 (51)		15 (52)	14 (48)	
CD8+ T cells (*n* = 213)	Low	38 (49)	39 (50)	1.0	14 (48)	14 (48)	1.0
	High	39 (51)	39 (50)		15 (52)	15 (52)	
FoxP3+ T cells (*n* = 209)	Low	35 (47)	38 (49)	0.9	11 (41)	18 (62)	0.2
	High	40 (53)	40 (51)		16 (59)	11 (38)	
CD20+ B cells (*n* = 212)	Low	36 (47)	37 (47)	1.0	16 (55)	13 (45)	0.6
	High	40 (53)	41 (53)		13 (45)	16 (55)	
CD68+ macrophages (*n* = 212)	Low	35 (45)	35 (45)	1.0	14 (52)	11 (37)	0.3
	High	42 (55)	43 (55)		13 (48)	19 (63)	
CD163+ macrophages (*n* = 208)	Low	37 (49)	37 (47)	1.0	13 (50)	12 (43)	0.8
	High	39 (51)	41 (53)		13 (50)	16 (57)	
Ki-67 (*n* = 212)	Low	41 (53)	36 (47)	0.5	13 (45)	15 (54)	0.6
	High	37 (47)	41 (53)		16 (55)	13 (46)	

*Data are number (%) or median (25 and 75 percentiles). NAC, neoadjuvant chemotherapy; EPP, extrapleural pneumonectomy; PD, pleurectomy/decortication; gC1qR, globular heads of the C1q receptor; Foxp3, forkhead box P3. Bold values indicate significant p-value*.

### Prognostic Implications of gC1qR Expression in Epithelioid MPM: gC1qR Expression Is Associated With Better OS in Patients Treated With Chemotherapy

**Figure 2:**
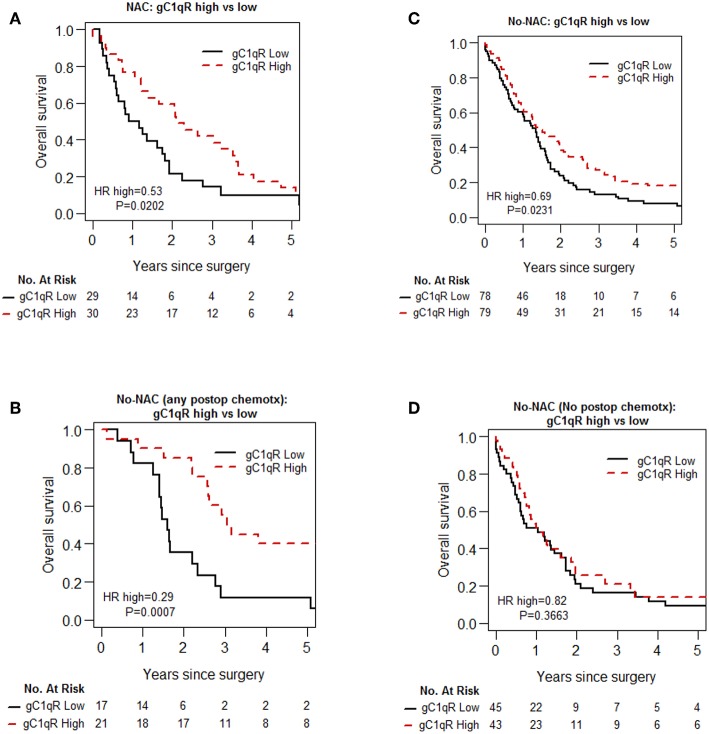
Kaplan-Meier analysis of overall survival of patients with epithelioid MPM in no-neoadjuvant chemotherapy (no-NAC) and neoadjuvant chemotherapy (NAC) cohorts. In patients with epithelioid MPM who received NAC, high gC1qR expression is associated with longer overall survival **(A)**. In patients who did not receive NAC (No-NAC,) high gC1qR expression is associated also with longer overall survival **(B)**, but the survival difference is limited to patients who received any post-operative chemotherapy **(C)**. No survival difference is noted in patients who did not receive post-operative chemotherapy **(D)**.

Univariable Cox proportional hazard analysis (Table 4) and Kaplan-Meier curves (Figure 2) for overall survival are shown. High gC1qR expression was associated with better overall survival in patients with epithelioid MPM in both NAC (Figure 2A; median OS, 25 vs. 11 months, *p* = 0.020) and No-NAC cohorts (Figure 2B; median OS, 18 vs. 16 months, *p* = 0.023). Interestingly, subanalysis of the no-NAC patient cohort reveals that high tumor gC1qR H score was significantly associated with longer overall survival in the No-NAC cohort in patients who received postoperative chemotherapy (Figure 2C), but not in patients who did not receive postoperative chemotherapy (Figure 2D).

**Table 4:** Univariable Cox proportional hazard analysis for overall death in patients with epithelioid MPM in no neoadjuvant chemotherapy(no-NAC) and neoadjuvant chemotherapy (NAC) cohorts.

		**Cohorts**	**No-NAC**			**NAC**		
**Variable**			**HR**	**95% CI**	***P***	**HR**	**95% CI**	***P***
Age (per 1 year increase)			1.01	0.99, 1.02	0.4	1.01	0.99, 1.04	0.3
Male (vs. female)			1.32	0.92, 1.90	0.13	1.86	1.02, 3.42	**0.045**
Smoking (vs. no smoking)			1.35	0.86, 2.11	0.2	0.95	0.52, 1.75	0.9
Asbestos (vs. no asbestos)			1.12	0.73, 1.71	0.6	1.38	0.77, 2.47	0.3
Procedure (vs. EPP)		PD	0.88	0.63, 1.23	0.5	1.08	0.60, 1.94	0.8
		Other	1.00	0.46, 2.18	1.0	1.14	0.35, 3.76	0.8
R2 (vs. R1)			1.16	0.73, 1.85	0.5	1.15	0.48, 2.75	0.7
Postoperative chemotherapy (vs. no chemotherapy)			0.60	0.40, 0.89	**0.012**			
p-Stage III/IV (vs. I/II)			2.20	1.52, 3.18	**<0.001**	1.14	0.65, 1.98	0.6
T category (vs. T1)		T2	2.32	1.14, 4.72	**0.020**	0.32	0.11, 0.98	**0.045**
		T3	3.12	1.53, 6.39	**0.002**	0.54	0.18, 1.60	0.3
		T4	2.59	1.01, 6.62	**0.047**	0.53	0.13, 2.15	0.4
N category (vs. N0)		N1	2.59	1.45, 4.60	**0.001**	0.66	0.09, 4.81	0.7
		N2	1.38	0.94, 2.02	0.10	0.98	0.47, 2.05	1.0
Pleomorphic positive (vs. negative)			1.79	1.13, 2.82	**0.013**	1.57	0.78, 3.16	0.2
Lymphatic invasion positive (vs. negative)			1.53	1.10, 2.11	**0.011**	1.55	0.88, 2.74	0.13
Vascular invasion positive (vs. negative)			1.97	1.34, 2.89	**0.001**	1.20	0.65, 2.22	0.6
CD3 high (vs. low)			1.08	0.78, 1.50	0.6	0.90	0.52, 1.54	0.7
CD4 high (vs. low)			0.75	0.54, 1.04	0.083	1.04	0.60, 1.78	0.9
CD8 high (vs. low)			0.73	0.52, 1.02	0.062	0.86	0.50, 1.48	0.6
CD20 high (vs. low)			0.71	0.51, 0.98	**0.039**	0.47	0.26, 0.84	**0.011**
FoxP3 high (vs. low)			0.91	0.66, 1.26	0.6	1.07	0.61, 1.88	0.8
CD68 high (vs. low)			1.28	0.92, 1.77	0.14	0.75	0.43, 1.31	0.3
CD163 high (vs. low)			1.06	0.76, 1.47	0.7	1.67	0.92, 3.02	0.092
Ki-67 high (vs. low)			2.24	1.58, 3.18	**<0.001**	1.03	0.59, 1.78	0.9
gC1qR high (vs. low)			0.69	0.50, 0.96	**0.029**	0.53	0.30, 0.91	**0.022**
CD4-gC1qR combination^*^	C*D4*	g*C1q*R						
	High	High	Ref					
	Low	Low	1.70	1.06, 2.75	**0.029**			
	Low	High	2.17	1.36, 3.45	**0.001**			
	High	Low	2.26	1.43, 3.55	**<0.001**			
Ki-67-gC1qR combination^*^	K*i6*7	g*C1q*R						
	Low	High	Ref					
	Low	Low	2.41	1.49, 3.90	**<0.001**			
	High	Low	3.08	1.85, 5.14	**<0.001**			
	High	High	4.04	2.42, 6.74	**<0.001**			

*Data are number (%) or median (25 and 75 percentiles)*.

* *Combination based on significant interactions between 2 variables in no NAC cohort. MPM, malignant pleural mesothelioma; NAC, neoadjuvant chemotherapy; EPP, extrapleural pneumonectomy; PD, pleurectomy/decortication; gC1qR, globular heads of the C1q receptor; Foxp3, forkhead box P3. Bold values indicate significant p-value*.

### Association Between gC1qR Expression and Ki-67 in Epithelioid MPM: Significant Prognostic Interaction Is Observed Between High Tumoral gC1qR Expression and Ki-67 Index in the No-NAC Patient Cohort

**Figure 3:**
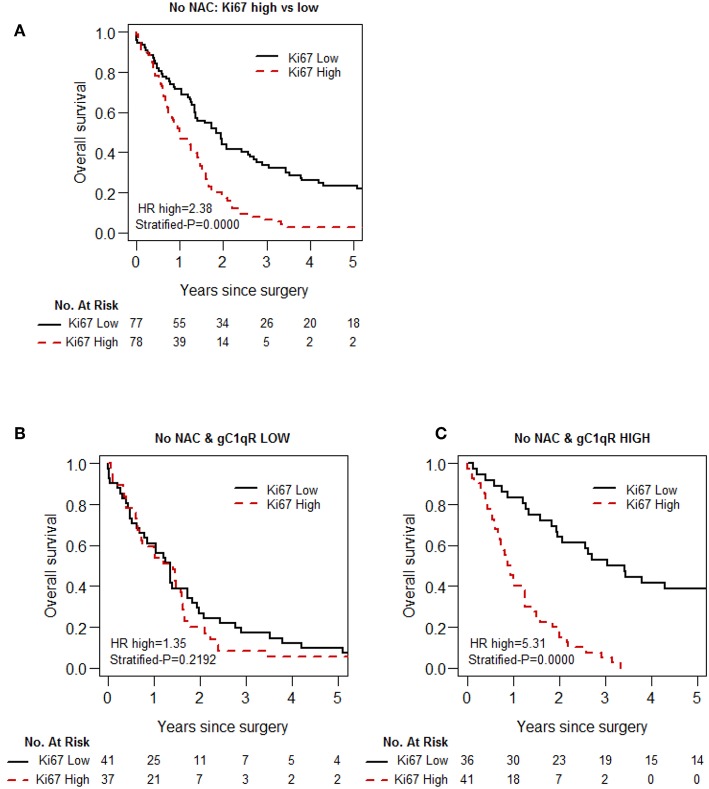
Kaplan-Meier analysis of overall survival of patients with epithelioid MPM, based on Ki-67 and gC1qR expression, in the No-NAC cohort. Whereas, high Ki-67 expression in epitheloid MPM is associated with shorter overall survival **(A)**, this prognostic effect of Ki-67 is blunted in patients in the No-NAC cohort whose tumors express low gC1qR H score **(B)**, and magnified in patients whose tumors express high gC1qR H scores **(C)**.

As the proliferative index of tumors, as measured by Ki-67 expression, is a widely investigated prognostic indicator for chemotherapy (39–41), the prognostic interaction between Ki-67 index and gC1qR expression was investigated. High Ki-67 expression was associated with poor overall survival, as confirmed in the no-NAC patient cohort (Table 4, Figure 3A). However, there was significant prognostic interaction between gC1qR and Ki-67 index (*p* = 0.001). Interestingly, when Ki-67 index was combined with gC1qR expression, no significant survival difference between high vs. low tumor Ki-67 index was noted in patients with low tumor gC1qR expression (Figure 3B) (HR 1.35, *p* = 0.219). In contrast, among patients with high tumor gC1qR expression, a marked decrease in overall survival was observed between high vs. low tumoral Ki-67 expression (Figure 3C) (HR 5.31, *p* < 0.001). No significant interaction between Ki67 and gC1qR was observed in the NAC group.

### Interaction Between gC1qR Expression and CD4 T Cell Infiltration in Epithelioid MPM: The Combination of High CD4 T Cell Infiltration and High Tumor gC1qR Expression Is Associated With Significantly Better Overall Survival

**Figure 4:**
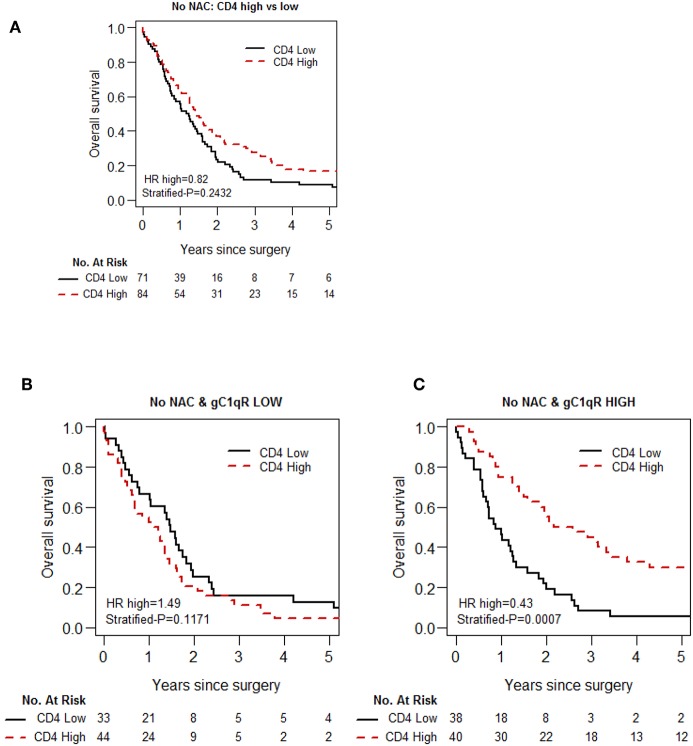
Kaplan-Meier analysis of overall survival of patients with epithelioid MPM in the No-NAC cohort based on tumoral CD 4 lymphocyte infiltration and gC1qR expression. Tumoral infiltration by CD 4 lymphocytes is not associated with a statistically significant increase in OS **(A)**, although a trend can be observed. Stratification of patients based on low **(B)** and high **(C)** gC1qR H scores, however, demonstrates a marked increase in overall survival in patients whose tumors demonstrate high gC1qR H scores and high CD4 lymphocyte infiltration.

Because gC1qR expression plays a role in immunomodulation (22), we explored the association between gC1qR expression and immune cell infiltration (CD3, CD4, CD8, FoxP3, CD20, CD68, and CD163) in epithelioid MPM. No significant association was observed between gC1qR expression and individual immune markers (Table 3). However, in the no-NAC cohort, the test of prognostic interaction between tumor infiltrating CD4 T cell score and gC1qR expression showed that there was a significant interaction between these two factors for overall survival (*p* = 0.002) (Table 4). Indeed, when CD4 T cell infiltration was stratified by gC1qR expression, the combination of high CD4 T cell infiltration and high tumor gC1qR expression was associated with significantly better overall survival compared to other combinations (Figure 4, Table 5). Whereas, CD4 infiltration was not significantly associated with overall survival among patients whose tumors expressed low levels of gC1qR (Figure 4B), high CD4 infiltration was associated with significantly improved overall survival among those with high tumor gC1qR H score (Figure 4C; HR 0.43, *p* = 0.0007). In univariable analysis, among patients with high tumor gC1qR expression, CD8 or CD20 lymphocyte infiltration were significantly associated with lower hazard of death (Table 5). No significant interaction between CD4 and gC1qR was observed in the NAC group.

**Table 5:** Univariable and multivariable analysis for any death in patients with epithelioid MPM in no NAC-cohort relative to gC1qR expression levels.

		**gC1qR Low (*****n*** **=** **78)**						**gC1qR High (*****n*** **=** **79)**					
		**Univariable**			**Final multivariable model**			**Univariable**			**Final multivariable model**		
		**Hazard Ratio**	**95%CI**	***P*-value**	**Hazard Ratio**	**95%CI**	***P*-value**	**Hazard Ratio**	**95%CI**	***P*-value**	**Hazard Ratio**	**95%CI**	***P*-value**
Age (per 1 year increase)		1	0.97, 1.02	0.8				1.02	1.00, 1.04	0.12			
Male gender (vs. female)		1.09	0.67, 1.78	0.7				1.65	0.95, 2.89	0.078			
Smoking (vs. non-smoking)		1.38	0.70, 2.69	0.4				1.34	0.73, 2.46	0.3			
Asbestos (vs. non-asbestos)		1.32	0.70, 2.50	0.4				1.11	0.61, 2.02	0.7			
Procedure (vs. EPP)	PD	0.86	0.53, 1.38	0.5				0.85	0.52, 1.37	0.5			
	Other	1.81	0.70, 4.69	0.2				0.48	0.12, 1.97	0.3			
R2 (vs. R1)		0.98	0.54, 1.77	0.9				1.27	0.58, 2.77	0.6			
Chemotherapy after surgery (vs. no chemotherapy)		0.83	0.47, 1.46	0.5				0.5	0.28, 0.87	**0.015**	0.54	0.29, 0.99	**0.047**
P stage III/IV (vs. I/II)		1.68	0.93, 3.02	0.083	1.41	0.76, 2.63	0.3	2.31	1.41, 3.79	**0.001**	2.99	1.66, 5.40	**0.0003**
T stage (vs. T1)	T2	1.14	0.27, 4.86	0.9				2.42	1.06, 5.50	**0.036**			
	T3	1.09	0.26, 4.58	0.9				4.13	1.72, 9.88	**0.001**			
	T4	1.13	0.23, 5.65	0.9				2.21	0.56, 8.70	0.3			
N stage (vs. N0)	N1	1.92	0.93, 3.98	0.079				3.51	1.36, 9.08	**0.009**			
	N2	0.97	0.55, 1.73	0.9				1.73	1.00, 2.97	**0.049**			
Pleomorphic positive (vs. negative)		2.86	1.43, 5.71	**0.003**	2.32	1.14, 4.74	**0.02**	1.48	0.78, 2.82	0.2			
Lymphatic invasion positive (vs. negative)		1.2	0.76, 1.91	0.4				1.9	1.19, 3.03	**0.007**			
Vascular invasion positive (vs. negative)		1.52	0.87, 2.64	0.14				2.6	1.46, 4.64	**0.001**			
gC1qR H score		1	1.00, 1.01	0.2				1	1.00, 1.01	0.6			
CD3 high (vs. low)		1.84	1.10, 3.08	**0.021**	1.64	0.96, 2.78	0.068	0.77	0.48, 1.23	0.3			
CD4 high (vs. low)		1.4	0.87, 2.26	0.2				0.46	0.28, 0.73	**0.001**	0.35	0.19, 0.64	**0.001**
CD8 high (vs. low)		1.01	0.64, 1.60	1				0.57	0.35, 0.92	**0.021**			
CD20 high (vs. low)		0.93	0.58, 1.48	0.7				0.55	0.34, 0.88	**0.013**			
FoxP3 high (vs. low)		0.93	0.58, 1.49	0.8				0.86	0.54, 1.37	0.5			
CD68 high (vs. low)		1.94	1.19, 3.16	**0.008**				1.01	0.63, 1.60	1			
CD163 high (vs. low)		1.46	0.91, 2.33	0.12				0.88	0.56, 1.40	0.6			
Ki67 high (vs. low)		1.27	0.79, 2.02	0.3				4.8	2.66, 8.64	**<0.0001**	7.13	3.36, 15.12	**<0.0001**

*Data are number (%) or median (25 and 75 percentiles). MPM, malignant pleural mesothelioma; NAC, neoadjuvant chemotherapy; EPP, extrapleural pneumonectomy; PD, pleurectomy/decortication; gC1qR, globular heads of C1q; forkhead box P3. Bold values indicate significant p-value*.

### Multivariable Analysis in NAC and No-NAC Patient Cohorts With Epithelioid MPM: High Tumoral gC1qR Expression Is Associated With Better OS in Patients Treated With Chemotherapy

Due to the relationships between gC1qR and Ki-67 expression as well as infiltrating immune cells in the no-NAC cohort, multivariable models were developed separately among patients with low gC1qR expression and high gC1qR expression to quantify differential impact of Ki67 and CD4 based on levels of gC1qR. As shown in Table 5 among patients with high tumor gC1qR H score, tumoral CD4 lymphocyte infiltration, p stage, and Ki-67 expression, were independently significant for reduced overall survival in multivariable cox proportional hazard analysis. Chemotherapy was independently associated with prolonged overall survival (HR = 0.54; 95% CI: 0.29–0.99; *p* = 0.047). In contrast, among patients with low tumor gC1qR H scores, only pleomorphic tumor phenotype was independently associated with worse overall survival in multivariable analysis.

Table 6 shows multivariable cox proportional hazard analysis for overall survival among the NAC cohort. The multivariable analysis included age, p stage, and aggressive pathologic features. High tumor gC1qR expression was an independent factor for overall survival only in the NAC patient cohort. Specifically, high gC1qR expression was associated with better overall survival compared to low gC1qR expression (HR = 0.54; 95% CI: 0.30–0.96; *p* = 0.037).

**Table 6:** Multivariable Cox proportional hazard analysis for overall death in patients with epithelioid MPM in no neoadjuvant chemotherapy (no-NAC) and neoadjuvant chemotherapy(NAC) cohorts.

	**Cohort**	**No-NAC**						**NAC**		
		**Multivariable model 1**			**Multivariable model 2**			**Multivariable model**		
		**HR**	**95% CI**	***P***	**HR**	**95% CI**	***P***	**HR**	**95% CI**	***P***
Age (per 1 year increase)								1.01	0.98, 1.03	0.7
Chemotherapy after surgery (vs. no chemotherapy)		0.52	0.34, 0.80	**0.003**	0.66	0.43, 1.02	0.059			
p-Stage III/IV (vs. I/II)		2.03	1.25, 3.28	**0.004**	2.36	1.49, 3.72	**<0.001**	1.35	0.75, 2.41	0.3
Pleomorphic positive (vs. negative)		1.98	1.07, 3.66	**0.029**	2.10	1.14, 3.86	**0.017**			
Vascular invasion positive (vs. negative)		1.57	0.99, 2.49	0.058						
CD20 high (vs. low)								0.46	0.25, 0.83	**0.010**
gC1qR high (vs. low)								0.54	0.30, 0.96	**0.037**

Data are number (%) or median (25 and 75 percentiles). ^*^Combination based on significant interactions between 2 variables in no NAC cohort. NAC, neoadjuvant chemotherapy; gC1qR, globular heads of the C1q receptor. Bold values indicate significant p-value.

## Discussion

This is the first study investigating the expression of gC1qR in MPM tumors, including epithelioid, biphasic, and sarcomatoid subtypes. The strengths of the study are: (1) large sample size, (2) the use of multiple TMA cores (6–9 cores for each patient), (3) inclusion of all 3 MPM histological subtypes, (4) long follow-up (median: 16 months), and (5) investigation of gC1qR expression in association with pathophysiologic characteristics, including infiltrating immune cells. Limitations of the study include evaluation of a surgical patient cohort and assessment bias inherent in the use of tumor cores rather than whole tissue sections.

gC1qR was overexpressed relative to normal pleura in all three histological subtypes of MPM (99%). Due to the small number of non-epithelioid subtypes, however, further analysis of gC1qR expression and pathophysiologic characteristics was limited to patients with epithelioid MPM. In this surgical patient cohort, high gC1qR expression correlated with better overall survival in patients who received NAC or any post-operative chemotherapy.

gC1qR expression in tumors of patients who received neither NAC or any postoperative chemotherapy did not significantly affect OS (HR 0.82, *p* = 0.3663). These findings are similar to those obtained using an independent data set (TCGA data-set) evaluated using Progene (http://genomics.jefferson.edu/proggene/index.php). Examination of this data set for gC1qR gene (C1QBP) expression in mesothelioma (*n* = 85) revealed that C1QBP expression was not correlated with OS [HR 1.08 (0.67–1.74, *p* = 0.755)]. This data set was not stratified for surgery or chemotherapy.

Chemotherapy is an important part of the trimodality treatment of MPM patients, albeit with a limited response rate. Moreover, there are no applicable biomarkers to select patients for chemotherapy (29, 30). Results from this study suggest that tumor gC1qR expression may be a prognostic indicator in surgical patients, since higher gC1qR expression was associated with longer survival in epithelioid MPM patients treated with either NAC or post operative chemotherapy. This observation is consistent with previous studies linking gC1qR expression with sensitivity to cisplatin treatment of cervical cancer cells and paclitaxel treatment of ovarian cancer cells *in vitro* (19, 20). Understanding the prognostic value of tumor gC1qR expression to identify MPM patients who are likely to benefit from chemotherapy pre or post surgery requires further investigation in prospective studies. Moreover, studies to understand the cell biology of gC1qR expression and cisplatin sensitivity are required. gC1qR expression may exert complex effects on tumor proliferation, as both cellular and extracellular gC1qR may play a role in immunomodulation via complement activation, recruitment of immune cells, and vascular permeability.

Ki-67 is a nuclear protein that is widely used as a tumor proliferation marker (40, 41). Our previous study showed that a high compared to low tumor Ki-67 index in patients with epithelioid MPM was associated with significantly worse median OS (38). Ki-67 expression also identifies a subset of patients with ER-positive breast cancer who could be sensitive to docetaxel treatment in the adjuvant setting (40). In the present study, univariable cox proportional hazard analysis confirmed that in patients who did not receive neoadjuvant chemotherapy, Ki-67 was associated with a higher hazard ratio for death. In addition, a high tumor Ki-67 index combined with a high tumor gC1qR H score, portended the worst OS, indicating the beneficial role of both Ki-67 and gC1qR as markers in stratifying benefit from chemotherapy. This finding is consistent with results obtained from the TCGA data set for mesothelioma which showed that tumors with C1QBP and Ki-67 upregulation had a worse OS [HR 2.44 (1.87–3.57, *p* = 4.3 × 10^−6^)] compared to high Ki-67 gene expression alone [HR 1.72 (1.37–2.17, *p* = 4.6 × 10^−6^)].

The apparent contradictory association between high gC1qR expression and worse OS in patients with tumors expressing a high Ki-67 index, compared to the overall favorable prognostic influence of high gC1qR expression in patients who undergo chemotherapy or whose tumors express high CD4 lymphocyte infiltration, is not well-understood. These observations underscore the complexity of tumor biology and support the hypothesis that MPM tumor progression is strongly influenced by both cellular and microenvironmental factors.

Inflammation and tumor infiltrating immune cells are thought to affect patient survival by influencing the host anti-tumor response. Our previous study showed that in a cohort of 175 patients with epithelioid MPM, those patients with high chronic stromal inflammatory responses had a better median overall survival than those with low chronic inflammatory responses (36, 42). Furthermore, we also found that high densities of tumoral CD4 and CD20 expressing lymphocytes were associated with better outcomes in epithelioid MPM (31). Interestingly, in the present study, high gC1qR expression combined with high CD4 staining in patients in the no- NAC cohort was associated with better OS in both univariable and multivariable analysis. Additional *in vitro* studies are required to understand the mechanism of this interaction, and how gC1qR may be involved in immune modulation. The complement system plays a pivotal role in regulation of innate and adaptive immunity. It has been shown that the binding of C1q to gC1qR on T cells will inhibit T cell proliferation (22, 43). Moreover, in chronic viral infection, direct binding of HCV core to gC1qR on CD4+ and CD8+ T cells led to impaired Lck/Akt activation and T cell function (23). This study investigated both gC1qR expression and CD4 T cell infiltration of tumors. Although gC1qR expression was not associated with differences in immune cell infiltration, patients bearing tumors with high gC1qR expression and high CD4 T cell infiltration had better OS than other combinations. Understanding the role of gC1qR expression in MPM tumor cell biology and its potential interactions with immune cell infiltration in the tumor microenvironment requires further study. The correlation of high gC1qR expression with lower tumor stage, and better OS in patients with epithelioid MPM differs from observations made in a variety of adenocarcinomas, in which increased gC1qR expression was associated with poor prognosis (15–18). As gC1qR is present in several cellular compartments, the interaction of cell surface and extracellular gC1qR with cellular and biochemical mediators in the microenvironment may play a significant role (12). Interestingly, C1q has been described in the microenvironment of epithelioid MPM, and to promote *in vitro* MPM cell proliferation and migration (44). These apparently contradictory observations demonstrate that *in vitro* models of tumor biology are not sufficiently complex to replicate *in vivo* pathogenesis. Direct immunohistochemical examination of patient tumors and clinicopathologic correlates contribute to a fuller understanding of the role of gC1qR and C1q in MPM.

Similar to C1q, hyaluronan has been demonstrated to play an important role in modulating cell proliferation and invasiveness in MPM (45). High levels of HA in the pleura have been reported to interfere with MPM tumor spread and are associated with favorable prognosis (46). HA binds a number of cellular receptors including CD44, RHAMM (receptor for HA-mediated motility), layilin, HARE (HA receptor for endocytosis), LYVE-1 (lymphatic vessel endocytic receptor), CD37, RHAMM/IHABP (intracellular HA-binding protein), P-32/gC1qR, and IHABP4 (47). In MPM, CD44-HA interactions modify cell signaling pathways triggering malignant cell migration and metastasis (45) and have been described as a possible docking/signaling molecule for gC1qR (48). The role played by HA binding to gC1qR in mesothelioma is not clear and needs further study. Of interest, similar to C1q and gC1qR expression by malignant cells and stroma (44), the effect of HA on tumor progression appears also to be dependent on cell type and tissue location (46).

In conclusion, this is the first study to investigate gC1qR expression and its pathophysiologic correlates in malignant pleural mesothelioma in a surgical patient cohort. The data demonstrate that gC1qR is overexpressed in tumors of all three histologic subtypes. In epithelioid MPM, gC1qR expression is prognostic for better overall survival in patients who received either neoadjuvant or post-operative chemotherapy. In patients who did not receive neoadjuvant chemotherapy, overall survival is positively influenced by the combination of high tumor gC1qR expression and high CD4 T-cell infiltration, and negatively impacted by the combination of high gC1qR expression and high Ki-67 index. Taken together, findings from this exploratory study support further investigation into the role of gC1qR as a potential new prognostic factor in patients with epithelioid MPM.

## Data Availability Statement

All datasets generated for this study are included in the manuscript/supplementary files.

## Ethics Statement

The studies involving human participants were reviewed and approved by Institutional Review Board of Memorial Sloan Kettering Cancer Center. The patients/participants provided their written informed consent to participate in this study.

## Author Contributions

EP, BG, and PA conceived the study. XL, EP, BG, and PA designed the experiments. XL, TE, RA, NC, MZ, EP, BG, and PA conducted the experiments. XL, EP, BG, KT, and PA carried out the data analysis. XL, EP, BG, and PA wrote the manuscript. EP and PA acquired funds and resources for the study and were responsible for project supervision. All authors were involved in data curation, reviewed, and edited the manuscript.

### Conflict of Interest

EP and BG receive royalties from the Stony Brook Research Foundation through the commercialization of gC1qR antibodies. The remaining authors declare that the research was conducted in the absence of any commercial or financial relationships that could be construed as a potential conflict of interest.

## Abbreviations

DAB: 3,3′-Diaminobenzidine
DC: dendritic cell
EPP: extrapleural pneumonectomy
Foxp3: forkhead box P3
gC1qR: globular heads of C1q receptor
HCV: hepatitis C virus
H&E: hematoxylin and eosin
IHC: immunohistochemistry
MPM: malignant pleural mesothelioma
NAC: neoadjuvant chemotherapy
OS: overall survival
P/D: pleurectomy and decortication
TMA: tissue microarrays.
